# Locomotor Health in Modern Broiler Chickens: Risk Factors, Major Disorders, Assessment Methods, and Implications for Welfare and Production Efficiency

**DOI:** 10.3390/ani16193051

**Published:** 2026-09-28

**Authors:** Emilia Lisiecka, Wiktoria Kościów, Monika Łukasiewicz Mierzejewska

**Affiliations:** Institute of Animal Science, Warsaw University of Life Sciences, Ciszewskiego 8, 02-786 Warsaw, Poland; emilia_lisiecka@sggw.edu.pl (E.L.); wiktoria_kosciow@sggw.edu.pl (W.K.)

**Keywords:** broiler chickens, locomotor system, skeletal health, lameness, gait assessment, animal welfare, production efficiency, precision livestock farming

## Abstract

Genetic selection for rapid growth, improved feed efficiency, and increased breast-muscle yield has substantially enhanced broiler production; however, it has also increased the functional demands placed on the locomotor system. This review critically examines locomotor health in modern broiler chickens in relation to welfare and production efficiency. Particular attention is given to the multifactorial interactions among genotype, growth rate, body weight and conformation, skeletal development, nutrition, physical activity, housing conditions, age, and infectious factors. Major locomotor disorders, including valgus–varus deformities, tibial dyschondroplasia, infectious arthritis and tenosynovitis, bacterial chondronecrosis with osteomyelitis, osteochondrosis, rickets, and fractures, are discussed in terms of their biological basis and welfare consequences. The review also evaluates current approaches to locomotor assessment, including gait scoring, standing ability, latency-to-lie testing, radiography, post-mortem examination, biomarkers, infrared thermography, force-plate analysis, and camera- and sensor-based monitoring. Evidence indicates that locomotor disorders should not be attributed solely to rapid growth but rather to insufficient coordination between increasing body mass and the adaptive capacity of skeletal and muscular tissues. Future strategies should integrate balanced genetic selection, precision nutrition, activity-promoting environments, and precision livestock farming technologies for early detection of impaired mobility. Such an integrated approach may help maintain high production efficiency while improving broiler welfare, supporting locomotor health, and reducing production losses.

## Simple Summary

Modern broiler chickens grow rapidly and achieve high production efficiency, but this can place substantial demands on the locomotor system. Leg disorders may reduce mobility, cause pain, limit access to feed and water, and negatively affect welfare and productivity. This review examines the main factors contributing to locomotor problems, including genetics, growth rate, body weight, nutrition, physical activity, housing conditions, age, and infections. It also summarizes common disorders and current methods used to assess locomotor health, from gait scoring to imaging, thermography, biomarkers, cameras, and sensors. Accordingly, maintaining locomotor health in modern broilers requires an integrated strategy combining genetic selection for skeletal robustness and functional mobility, precision nutrition matched to growth trajectory, housing conditions that promote physical activity, and early detection of locomotor impairment using validated monitoring technologies.

## 1. Introduction

Broiler chicken production is currently one of the most intensive and technologically advanced sectors of global animal production. Its importance stems from the short production cycle, rapid growth rate, highly efficient feed utilization, and the ability to provide a relatively inexpensive source of animal protein. However, the modern broiler is not only an example of production success but also the result of many years of intensive genetic selection, supported by advances in nutrition, veterinary prevention, and housing technology [1,2,3].

Comparisons among broiler lines originating from different breeding eras illustrate the magnitude of these changes. Since the mid-twentieth century, growth rate has increased more than fourfold, accompanied by a substantial improvement in feed efficiency and an increase in breast-muscle yield [4,5]. These advances have generated major production benefits, but they have also revealed biological limitations associated with uneven development of individual physiological systems. Of particular importance is the locomotor system, whose development is not always fully synchronized with the rapid accretion of muscle mass. Bones, joints, cartilage, tendons, and limb muscles must adapt within a short period to rapidly increasing loads, which may exceed their adaptive capacity [1,6,7].

Locomotor problems in broilers are not a new phenomenon. Kestin et al. (1992) already reported a high prevalence of gait abnormalities and differences among genotypes, and subsequent studies conducted in large commercial populations confirmed the considerable scale of the problem [8,9]. The fact that similar disorders have been reported for several decades indicates that, despite substantial advances in breeding, nutrition, and production technology, they have not been completely eliminated.

Locomotor disorders in broilers are multifactorial and should not be attributed to rapid growth alone. Their development reflects complex interactions among genotype, growth trajectory, skeletal maturation, nutrition, physical activity, housing conditions, and infectious and inflammatory factors [1,7,9]. From a welfare perspective, the consequences of locomotor disorders extend far beyond abnormal gait alone. Pain and reduced mobility may impair access to feed and water, restrict the performance of natural behaviors, and increase the risk of contact lesions and culling. The problem therefore has simultaneous health, welfare, production, and economic dimensions.

The future of broiler production should not be based on a simple trade-off between high performance and welfare. A more appropriate objective is biologically sustainable productivity, in which rapid growth remains functionally coordinated with skeletal and muscular development. This includes selection that accounts for gait and bone quality, precision nutrition, environments that promote activity, and the use of precision livestock farming technologies for the early detection of declining mobility [10,11,12].

The aim of this review is to critically evaluate the available evidence on the determinants of locomotor health in modern broiler chickens, with particular emphasis on the strength, consistency, and limitations of reported associations between genotype, growth, skeletal development, nutrition, physical activity, environmental conditions, and production outcomes.

### Methods

This review was conducted as a structured narrative review of the scientific literature concerning locomotor and skeletal health in modern broiler chickens. The literature search was initiated in January 2026 and updated during manuscript preparation using three electronic databases: Web of Science, Scopus, and PubMed. The search focused on four main areas: broiler chickens, locomotor and skeletal health, determinants and risk factors, and methods of locomotor assessment.

The principal search terms included “broiler”, “broiler chicken*”, “locomotor health”, “locomotor disorder*”, “leg disorder*”, “leg weakness”, “lameness”, “gait”, “walking ability”, “skeletal health”, “bone development”, “bone quality”, “growth rate”, “body weight”, “genetic*”, “genotype”, “nutrition”, “physical activity”, “stocking density”, “housing condition*”, “environmental enrichment”, and “heat stress”. Additional targeted searches were performed for individual locomotor disorders, including “tibial dyschondroplasia”, “valgus-varus deformity”, “arthritis”, “tenosynovitis”, “bacterial chondronecrosis”, “osteomyelitis”, “osteochondrosis”, “rickets”, and “fracture*”, as well as for assessment methods such as “gait score”, “latency-to-lie”, “radiography”, “infrared thermography”, “force plate”, “accelerometer”, “computer vision”, and “sensor-based monitoring”. A representative core search combination was: (“broiler chicken” OR broiler) AND (“locomotor health” OR “locomotor disorder*” OR “leg disorder*” OR “leg weakness” OR lameness OR gait OR “walking ability” OR mobility OR “skeletal health” OR “bone quality”)*. Search syntax was adapted to each database.

The electronic database searches identified 532 records: 168 in Web of Science, 221 in Scopus, and 143 in PubMed. To identify additional relevant literature, backward and forward citation searching of key primary studies, reviews, meta-analyses, and authoritative scientific sources was also performed, yielding 420 additional records. Thus, the initial literature pool comprised 952 records. After removal of 480 duplicate or overlapping records, 472 unique publications were screened by title and abstract.

Studies were considered eligible when they addressed locomotor or skeletal health in broiler chickens and provided relevant information on genetic, nutritional, environmental, pathological, welfare, production, or methodological aspects of locomotor function. The review focused primarily on English-language, peer-reviewed original studies, while relevant reviews, meta-analyses, and authoritative scientific assessments were included when they provided broader mechanistic or historical context. No fixed lower publication-year limit was applied because several earlier studies remain fundamental to the understanding of broiler locomotor disorders.

Following title and abstract screening, 325 records were excluded as being outside the scope of the review, leaving 147 publications for full-text assessment. Of these, 90 were excluded because they did not provide sufficiently relevant information on broiler locomotor or skeletal health or substantially overlapped with evidence more appropriately represented by other sources. Ultimately, 57 publications were retained for direct inclusion in the qualitative synthesis.

The included evidence was organized according to major thematic areas: determinants and risk factors for locomotor dysfunction; major locomotor and skeletal disorders; welfare and production consequences; and conventional and emerging methods of locomotor assessment. Evidence was synthesized qualitatively, taking into account study design, broiler genotype, age, growth characteristics, housing and nutritional conditions, outcome definitions, and assessment methods. Formal quantitative pooling was not performed because of substantial heterogeneity among studies in populations, experimental designs, interventions, diagnostic criteria, and reported outcomes.

## 2. Causes of Locomotor System Problems

Locomotor disorders in modern broilers result from interactions among genetic, developmental, nutritional, biomechanical, environmental, and infectious factors. Rather than acting independently, these factors modify the ability of the musculoskeletal system to adapt to the rapid changes in body mass and body conformation that occur during the short production cycle. The following sections discuss the major contributing factors individually while emphasizing their biological interdependence.

### 2.1. Genetic Factors

Genetic background is an important determinant of locomotor health in modern broilers, although its effects cannot be separated from growth rate, body conformation, and management conditions. Early evidence demonstrated substantial differences among genotypes in susceptibility to leg weakness, supporting a hereditary component of locomotor impairment [8]. Subsequent studies comparing fast- and slower-growing lines generally reported poorer gait, lower activity, and a greater occurrence of leg problems in birds selected for rapid growth [1,9]. However, these associations should not be interpreted as evidence that genetic selection for growth alone directly causes locomotor disorders. Selection has simultaneously altered several correlated traits, including body weight, breast-muscle yield, body proportions, and load distribution, which may collectively increase biomechanical demands on the osteoarticular system [3].

Therefore, the relevant breeding trade-off is not simply between growth and leg health, but between continued improvement in production traits and the capacity to maintain structural and functional robustness of the locomotor system. Contemporary breeding strategies increasingly incorporate functional traits such as gait quality, bone strength, limb conformation, and physical activity alongside production performance [13,14,15]. Nevertheless, the extent to which improvements in these traits can offset the biomechanical consequences of continued selection for rapid growth and high breast yield remains an important area for further evaluation.

Genetic analyses further demonstrate that skeletal traits have an additive genetic component and can respond to selection. González-Cerón et al. (2015) reported substantial variation in heritability among bone-quality traits and identified both favorable and unfavorable genetic relationships between growth and indicators of skeletal integrity [13]. This indicates that the relationship between production potential and skeletal robustness is trait-specific rather than representing a simple antagonism between growth and bone quality.

Long-term breeding data provide further evidence that genetic improvement of leg health is feasible. Kapell et al. (2012) demonstrated substantial improvement in several leg-health traits over 25 years of selection in commercial purebred broiler lines, despite low-to-moderate heritabilities and partially unfavorable genetic correlations with body weight [16]. More recent analyses showed that gait score is also heritable, with estimates ranging from 0.14 to 0.24, and that its genetic correlations with body weight and breast-muscle percentage are unfavorable but moderate [16,17]. Importantly, both studies indicate that locomotor and production traits can be improved simultaneously within balanced multi-trait breeding strategies.

Comparisons of faster- and slower-growing commercial genotypes also indicate differences in activity, leg health, and broader welfare outcomes [14,15]. Therefore, contemporary breeding objectives should not focus simply on reducing growth potential but rather on improving the balance among growth, feed efficiency, breast yield, skeletal integrity, and functional mobility. Although modern breeding programs increasingly incorporate leg-health and welfare-related traits, the extent to which genetic improvement can fully compensate for the biomechanical consequences of continued selection for high growth rate and breast-muscle yield remains incompletely resolved.

### 2.2. Rapid Growth Rate

Rapid growth is consistently associated with an increased risk of locomotor impairment in broilers, but the available evidence suggests that growth rate should not be considered an isolated causal factor. Rather, the risk appears to arise when the rate of body-mass accretion exceeds the capacity of bones, joints, cartilage, and tendons to mature and adapt to increasing mechanical loads [7,18]. This imbalance may contribute to limb deformities, abnormal cartilage development, and poorer gait quality, particularly during later stages of rearing when body weight increases rapidly and spontaneous physical activity declines.

Importantly, reduced activity may function both as a consequence and as a potential amplifier of locomotor dysfunction, creating a bidirectional relationship between body mass, movement, and musculoskeletal adaptation. Therefore, strategies aimed at improving locomotor health should not focus solely on reducing growth rate. A more appropriate approach is to optimize the growth trajectory so that skeletal and connective-tissue development remain better synchronized with increasing body mass and production performance [19,20,21].

### 2.3. Body Weight and Morphological Changes

Body weight and body conformation are important determinants of locomotor function in modern broilers, although their effects are closely interrelated with growth rate, genotype, and skeletal development. Selection for increased breast-muscle yield has altered body proportions by increasing the relative mass of the anterior body and shifting the center of gravity forward. Together with relatively short limbs, these changes modify gait biomechanics, increase mechanical loading of the joints, and may increase the energetic cost of locomotion [7].

However, high body weight should not be regarded as an independent cause of locomotor pathology. Rather, the available evidence suggests that its adverse effects become particularly important when body conformation and skeletal capacity are insufficient to accommodate the increasing mechanical load. Altered posture and gait may consequently promote fatigue and reduce spontaneous activity, which may further compromise musculoskeletal adaptation. Thus, the relevant issue is not final body weight alone, but the interaction between body mass, body proportions, limb geometry, and the functional capacity of the supporting apparatus. Future breeding strategies should therefore consider these biomechanical and functional traits alongside body weight and breast-muscle yield.

### 2.4. Reduced Physical Activity

Reduced physical activity may be both a cause and a consequence of impaired locomotor function in broilers [18,22,23]. Fast-growing birds spend a substantial proportion of time sitting or lying, owing to high body weight, the energetic cost of locomotion, and environmental conditions. Regular loading of the limbs during movement, however, stimulates the development of bones, muscles, and joints. Insufficient activity may therefore limit their functional adaptation to increasing body mass and create a vicious cycle: growth restricts movement, inactivity weakens the musculoskeletal system, and impaired function further reduces activity [18,22]. In advanced cases, reduced mobility may hinder access to feed and water, directly compromising welfare [3,6]. An important preventive strategy is therefore not only the early detection of gait changes, but also the design of environments that stimulate birds to be active before clinical locomotor disorders appear [23,24]. Current evidence therefore supports a bidirectional relationship rather than a simple causal pathway: reduced activity may both reflect existing locomotor impairment and contribute to further deterioration of musculoskeletal function [18,22,24]. This makes it difficult to determine from cross-sectional observations whether inactivity is primarily a cause, a consequence, or both.

### 2.5. Nutritional Factors

Adequate nutrition is a fundamental prerequisite for harmonious skeletal development in broilers, and its importance increases as selection for rapid growth becomes more intensive. Earlier studies demonstrated that deficiencies of calcium, phosphorus, and vitamin D3 lead to impaired bone mineralization, rickets, and increased susceptibility to limb deformities [6,25]. In modern broilers, however, the problem is not limited to deficiency of a single nutrient; rather, it concerns the balance between the requirements of a rapidly growing organism and its ability to utilize nutrients supplied in the diet.

Calcium and phosphorus are the principal constituents of hydroxyapatite, whereas vitamin D3 regulates their absorption and bone metabolism. Not only the dietary levels of these nutrients but also their ratios and bioavailability are important. Studies have shown that vitamin D3 requirements may depend on calcium and available phosphorus concentrations, while disturbances in vitamin D metabolism may increase susceptibility to tibial dyschondroplasia [25,26]. Mineral source and bioavailability are therefore particularly relevant because diets with similar formulated nutrient concentrations may differ in the proportion of minerals that can actually be utilized for skeletal development.

Skeletal development also depends on adequate nutrient density and amino acid supply, because the organic bone matrix, muscle tissue, and connective structures must develop in parallel with mineral deposition. Amino acids involved in the formation of the organic bone matrix and trace elements, including Mn, Zn, and Cu, which participate in chondrocyte differentiation, osteoblast activity, and collagen formation, are therefore important [27]. In addition, interactions among dietary components can substantially modify nutrient availability. In particular, phytate reduces phosphorus availability, whereas exogenous phytase can improve phosphorus utilization, tibial mineralization, and bone strength; however, the magnitude of this response varies with bird age, strain, dietary phosphorus source, and duration of phytase supplementation [28]. Dietary electrolyte balance may also influence bone development through its effects on acid–base and mineral homeostasis. Experimental evidence indicates that different electrolyte-balance levels can modify femoral dimensions and mechanical properties, although these responses are context-dependent and should not be interpreted independently of overall diet composition [29].

Current evidence increasingly points to relationships among nutrition, gastrointestinal function, and bone health, expanding the classical concept of mineral metabolism to include the so-called gut–bone axis [30]. The high performance of modern broilers therefore leaves a relatively narrow margin for nutritional error. A diet sufficient for a slower-growing genotype may not ensure optimal mineralization in birds with greater growth potential. Future strategies should therefore move away from simply increasing mineral concentrations and toward precision nutrition tailored to age, genotype, and growth trajectory, while also accounting for Ca and P bioavailability, vitamin D3 metabolites, nutrient density, amino acid balance, and interactions among dietary components [31].

Although the importance of adequate Ca, P, and vitamin D3 supply is well established, the evidence is less conclusive regarding the extent to which nutritional optimization can compensate for the high skeletal demands imposed by modern growth rates. Nutritional effects should therefore be interpreted in the context of genotype, growth trajectory, nutrient bioavailability, and interactions among dietary components.

### 2.6. Metabolic and Developmental Disorders

Metabolic and developmental disorders are important components of the pathogenesis of locomotor problems in broilers because they may disrupt normal formation, mineralization, and remodeling of bone and cartilage. Their consequences include reduced strength of supporting structures, limb deformities, and increased susceptibility to injury, ultimately impairing gait quality [6]. These problems rarely occur independently. They most commonly result from the interaction of rapid growth, high metabolic demand, and insufficient skeletal maturation. In fast-growing broilers, body-mass gain may outpace normal mineralization and remodeling of the growth plate, favoring the formation of poorly mineralized areas and limb deformities [27]. Metabolic disorders should therefore be regarded not as isolated entities but as manifestations of an imbalance between the bird’s growth potential and the adaptive capacity of the skeletal system. Prevention should consequently include simultaneous control of growth trajectory, mineral nutrition, and normal bone development.

### 2.7. Infectious Factors

Infectious agents can cause inflammation of joints, tendon sheaths, bone, and bone marrow, resulting in pain, swelling, restricted mobility, and lameness [6,23]. The major pathogens associated with locomotor disorders in broilers include *Mycoplasma synoviae*, avian reoviruses, and bacteria such as *Escherichia coli*, *Staphylococcus* spp., and *Salmonella* spp. [23]. Infection, however, is not always the primary cause of the problem. Rapid growth, mechanical overload, and microdamage to cartilage and growth plates may create sites predisposed to bacterial colonization, blurring the distinction between developmental and infectious disorders. Bacterial chondronecrosis with osteomyelitis (BCO) is a notable example in which mechanical and infectious factors jointly contribute to disease pathogenesis. Reducing infectious causes of lameness therefore requires not only effective biosecurity and pathogen control but also mitigation of predisposing factors related to excessive loading of an immature skeletal system.

### 2.8. Age

Age is an important factor modulating the severity of locomotor disorders in broilers because body weight and musculoskeletal loading increase rapidly over time. Problems that may have little functional significance in young birds can lead to marked gait deterioration, lameness, and reduced activity later in the rearing period [7,32]. Age should, however, be viewed not as an independent cause but as a factor that reveals the cumulative effects of earlier metabolic, mechanical, and environmental challenges. During the final rearing phase, increasing body mass may exceed the compensatory capacity of bones, joints, and muscles, particularly when developmental abnormalities or reduced activity have occurred earlier. Prevention of locomotor problems should therefore begin early in life, before increasing body weight exposes functional abnormalities.

### 2.9. Environmental and Housing Conditions

Environmental and housing conditions can substantially modify the expression of genetic, metabolic, and biomechanical predispositions to locomotor disorders in broilers. Stocking density is particularly important because increasing density reduces the space available for unrestricted movement and may decrease spontaneous activity. However, the relationship between stocking density and leg health is not linear and is influenced by body weight, genotype, litter quality, temperature, ventilation, and access to feed and water [6,7,27]. Consequently, reducing stocking density alone does not necessarily improve locomotor outcomes when other environmental conditions remain unfavorable [33,34,35,36].

Litter quality represents another important component of the housing environment. Wet or compacted litter increases the risk of footpad dermatitis and hock lesions, which may reduce movement through pain. At the same time, birds with impaired locomotor function spend longer periods sitting or lying and are therefore more exposed to poor litter conditions. This indicates a bidirectional relationship in which deteriorating litter quality may contribute to reduced mobility, while reduced mobility itself increases exposure to damaging litter conditions [7,18].

Lighting programs also influence locomotor activity by regulating periods of activity and rest. Appropriately designed light–dark schedules may promote movement, particularly during early rearing, when mechanical loading contributes to the development of bones and muscles [18,22]. Nevertheless, lighting should not be considered in isolation, because its effects on activity depend on other environmental factors, including stocking density, resource distribution, and opportunities for exploration. Studies on environmental complexity and elevated structures suggest that enrichment can increase activity and may improve some indicators of leg condition or walking ability, although effects on skeletal traits are not always consistent across studies [37,38,39,40,41].

Access to feeders and drinkers is also important, particularly for birds with declining mobility. Resources should remain readily accessible, because increased walking distance or competition may further disadvantage birds with impaired locomotor capacity [6,27]. At the same time, spatial distribution of resources and environmental enrichment may be used to stimulate voluntary movement in birds that remain capable of normal locomotion.

Heat stress constitutes an additional environmental challenge, particularly in fast-growing broilers with high metabolic heat production and limited capacity for heat dissipation. Prolonged exposure to high ambient temperatures may reduce feed intake, alter mineral and hormonal homeostasis, and intensify oxidative and inflammatory responses, potentially affecting bone development and mechanical properties. However, these effects are likely to depend on the severity and duration of heat exposure, bird age, nutritional status, and genotype and should therefore not be interpreted independently of the broader production environment. Heat stress constitutes an additional environmental challenge, particularly in fast-growing broilers with high metabolic heat production and limited capacity for heat dissipation. Prolonged exposure to high ambient temperatures may reduce feed intake, alter mineral and hormonal homeostasis, and intensify oxidative and inflammatory responses, potentially affecting bone development and mechanical properties. Experimental studies have demonstrated that heat stress can reduce tibial mineral content, bone mass, and material properties, while high rearing temperatures may also decrease bone mineralization, femoral breaking strength, and tibiotarsal rigidity [42,43]. However, these effects are likely to depend on the severity and duration of heat exposure, bird age, nutritional status, and genotype and should therefore not be interpreted independently of the broader production environment.

Thus, the available evidence supports an integrated environmental-management approach in which stocking density, litter quality, microclimate, lighting, resource distribution, and opportunities for activity are considered jointly rather than individually [39,40,41].

Overall, environmental factors appear to act mainly as modifiers of locomotor risk rather than as isolated causes. Their effects are highly context-dependent and may vary with genotype, body weight, stocking density, litter condition, temperature, and access to resources [6,7,27,33,34,35,36].

#### Interactions Among Factors

Locomotor disorders in broilers arise from interacting rather than independent risk factors. Genetic predisposition and rapid growth may increase baseline susceptibility, while body weight, nutrition, physical activity, environmental conditions, and infection can modify the expression and severity of locomotor dysfunction [6,7]. Importantly, these relationships are bidirectional: reduced activity may both result from and contribute to musculoskeletal impairment, while poor environmental conditions can amplify the effects of existing biological vulnerability. Thus, locomotor disorders should be interpreted as the outcome of a network of mutually reinforcing influences rather than a single causal pathway [22,27].

## 3. Characteristics of the Most Common Locomotor Disorders

The major locomotor disorders observed in broiler chickens differ in their predominant pathogenesis, anatomical location, age of occurrence, clinical expression, and welfare consequences. To facilitate direct comparison among these conditions, their key characteristics and principal diagnostic approaches are summarized in Table 1. Detailed mechanisms, clinical features, and supporting evidence are discussed in the corresponding subsections below.

### 3.1. Valgus–Varus Deformity (VVD)

VVD are characteristic developmental disorders of broiler limbs involving abnormal alignment of the distal tibiotarsus. In valgus deformity, the limb deviates outward, whereas in varus deformity it deviates inward, producing an X-shaped or O-shaped limb configuration, respectively. Lesions may also involve the tarsometatarsus and disrupt normal limb biomechanics. VVD has been reported more frequently in males, and varus deformity may cause severe lameness at an early age, including as a result of displacement of the gastrocnemius tendon [6].

The significance of VVD extends beyond the anatomical deformity itself. Alteration of the limb axis changes the distribution of forces acting on joints and bones, increasing the risk of overload and secondary gait deterioration. The progression of lesions as the bird grows indicates an interaction between developmental predisposition and rapidly increasing body mass. VVD can therefore be regarded as an example of a disorder in which abnormal limb development becomes clinically apparent only under increasing biomechanical load. Prevention should include both selection for correct limb conformation and management of growth rate and environmental conditions that promote physical activity.

### 3.2. Tibial Dyschondroplasia (TD)

TD is one of the most important disorders of long-bone development in broilers and a major cause of impaired locomotor function. It is characterized by accumulation of unmineralized cartilage within the growth plate of the tibiotarsus as a result of disturbed chondrocyte maturation, matrix mineralization, and normal ossification [6,27]. In severe cases, bone structure becomes markedly weakened, leading to deformity, unstable gait, and reluctance to move, and birds may use their wings for additional support [23].

Importantly, milder forms of TD may remain poorly evident clinically despite the presence of structural abnormalities. Earlier studies already indicated a high prevalence of subclinical lesions, demonstrating that assessment based solely on visible lameness may underestimate the true scale of the problem [6]. TD clearly illustrates the biological cost of rapid growth: the rate of bone elongation and cartilage proliferation may exceed the capacity for vascularization, mineralization, and remodeling. Reducing TD therefore requires not only appropriate mineral nutrition but also better synchronization of growth rate with growth-plate maturation [44].

### 3.3. Infectious Arthritis and Tenosynovitis

Infectious arthritis and tenosynovitis may occur in acute, chronic, or subclinical forms and can result in pain, swelling, and restricted joint mobility. Lesions most commonly involve the hock region and structures associated with the digital flexor and gastrocnemius tendons. Microorganisms associated with the disease include: *Staphylococcus aureus*, *Escherichia coli*, *Salmonella* spp., *Pasteurella multocida*, *Erysipelothrix rhusiopathiae*, and *Pseudomonas* spp. [6]. In the study by Pedersen et al. (2020), such lesions were identified in 11.9% of 1429 examined birds [45].

From a welfare perspective, the importance of the disease derives primarily from pain and the progressive restriction of mobility. Infection itself, however, should not be analyzed independently of environmental conditions and limb status. Skin injuries, tissue microdamage, and overload of the locomotor apparatus may facilitate pathogen entry and development of inflammation. Prevention, therefore, cannot be limited to biosecurity alone. Mechanical damage must also be minimized, good litter quality maintained, and deteriorating gait identified rapidly. The disease thus represents an example of interaction between infectious and environmental–mechanical factors.

### 3.4. Bacterial Chondronecrosis with Osteomyelitis (BCO)

BCO is one of the most important causes of severe lameness in modern broilers. Lesions primarily affect cartilage, growth plates, and bone tissue in the proximal femur and tibia. Hematogenous bacteria may colonize damaged areas, leading to inflammation, chondrocyte necrosis, and weakening of bone structure. *Staphylococcus aureus* is most commonly implicated, although *E. coli*, *Salmonella* spp., and other staphylococci have also been isolated [6,23].

BCO is a particularly informative example of a disease in which the boundary between infectious and biomechanical pathology becomes blurred. Rapid growth and high loading of immature osteochondral structures promote microdamage that may subsequently provide sites for bacterial colonization [23]. Clinically, birds may show lameness, reluctance to move, use of the wings for support, and, in advanced cases, loss of the ability to walk. Historical and contemporary evidence indicates that BCO remains an important problem in commercial flocks. Prevention should therefore combine infection control with reduction in mechanical overload and early detection of locomotor changes.

### 3.5. Osteochondrosis

Osteochondrosis is a disorder of endochondral ossification occurring primarily in young, rapidly growing organisms. Its etiology is multifactorial and includes genetic predisposition, rapid growth, anatomical conformation, trauma, and nutritional factors. In normally developing bone, growth-plate cartilage undergoes progressive mineralization and vascularization and is replaced by bone tissue. In osteochondrosis, this process is disrupted, including through vascular disturbances and hypoxia, leading to the formation of necrotic foci and persistence of abnormal cartilage [46,47].

In broilers, lesions have been described in the cervical and thoracic vertebrae and in the femoral epiphysis [48]. Consequences may include a shortened stride, reluctance to walk, increased time spent sitting, and pain. Osteochondrosis demonstrates that rapid growth may disrupt not only mineralization but also normal vascularization of developing cartilage. Its pathogenesis therefore cannot be reduced to mineral deficiencies; rather, it reflects disruption of the entire process of growth-plate maturation and remodeling.

### 3.6. Rickets

Rickets is a metabolic bone disease associated primarily with deficiency or impaired availability of calcium, phosphorus, and vitamin D3. Inadequate supply, inappropriate ratios, or impaired absorption of these nutrients results in insufficient mineralization of the developing skeleton. In severe cases, bones become soft and susceptible to deformation and fracture; however, subclinical forms that are not visible during routine flock observation may also be of particular production importance.

Studies conducted as early as the 1990s reported rickets-like lesions in young broilers, demonstrating that impaired mineralization has accompanied intensive production for several decades. Rickets may also coexist with tibial dyschondroplasia, although the nature of this relationship has not been fully clarified [6]. Today, rickets should be considered not only as a simple consequence of mineral deficiency but also as a potential result of a mismatch between nutrient supply and utilization and the exceptionally rapid development of the skeleton. Prevention therefore requires precise balancing of Ca, P, and vitamin D3 according to genotype and stage of growth.

### 3.7. Fractures

Fractures represent the final manifestation of insufficient bone capacity to withstand the biomechanical forces acting upon it. In broilers, they may result from trauma, overload of the rapidly growing skeleton, or occur secondary to other pathologies such as tibial dyschondroplasia or BCO [23,27]. Their prevalence may depend on the housing system and environmental enrichment; Pedersen et al. (2020) reported values ranging from 0.67 to 6.90% [45].

The significance of fractures extends beyond the immediate loss of bone integrity. They reflect the relationship among cortical and trabecular bone quality, mineralization, bone geometry, and the magnitude of loading imposed on the limb. Therefore, increasing bone mineral content alone does not necessarily ensure adequate mechanical resistance. Risk assessment should also include biomechanical properties such as breaking strength, stiffness, and bone geometry. From a welfare perspective, fractures cause severe pain and reduced mobility, while from a production perspective they increase the risk of culling and economic loss. Their occurrence can therefore be regarded as an endpoint indicating loss of balance between body loading and skeletal strength.

### 3.8. Consequences of Locomotor Disorders

Locomotor disorders are among the most serious welfare problems in broilers because they can cause pain, restrict activity, and impair basic behaviors, including movement, resting, and access to feed and water [18,27]. As lameness becomes more severe, birds spend more time lying and modify their feeding behavior, including reducing the frequency of visits to feeders, although total feeding time may not necessarily decline proportionally. Consequently, the relationship between locomotor impairment and feed intake is not always straightforward and may depend on the severity and duration of lameness.

From a production perspective, locomotor impairment may affect performance through several pathways. Reduced mobility can impair access to feed and water, alter feeding patterns, reduce growth potential, and increase the probability of culling and mortality. At flock level, poorer locomotor condition has also been associated with production and slaughter-related outcomes. In commercial broiler flocks, impaired gait has been linked with early mortality and slaughterhouse indicators, while increasing gait impairment has been associated with a higher proportion of carcasses condemned as unfit for human consumption [49,50]. These observations indicate that locomotor dysfunction may generate losses not only during rearing but also at processing.

However, the relationship between leg health and production efficiency is complex. Genotypes or management strategies associated with improved locomotor health do not necessarily maximize short-term growth rate or feed conversion. For example, slower-growing broilers generally show better locomotor outcomes and lower lameness-related culling, but they may require more feed and a longer rearing period to reach comparable slaughter weights, resulting in less favorable conventional feed-efficiency indices [14]. Conversely, management interventions that improve activity and leg condition may, under some commercial conditions, simultaneously reduce culling and mortality without compromising growth performance. Therefore, improvements in locomotor health should be evaluated using an integrated set of indicators rather than body weight or feed conversion ratio alone.

Historical prevalence data provide an important reference point for evaluating the scale of locomotor problems in broiler production. Knowles et al. (2008), analyzing approximately 51,000 broilers, reported gait abnormalities in 27.6% of birds, while 3.3% showed severely impaired walking ability [9]. These values should, however, be interpreted as historical estimates and should not be assumed to represent the prevalence observed under current commercial conditions. More recent commercial-flock studies reported moderate-to-severe gait impairment in 24.6% of 5900 Ross 308 broilers from 59 commercial flocks (Kittelsen et al., 2017) and in approximately 19% of 7500 broilers from 50 flocks (Granquist et al., 2019) [49,50]. Nevertheless, these values should not be interpreted as universal contemporary prevalence estimates, because reported frequencies depend on genotype, age, production system, management conditions, and gait-scoring methodology. Direct comparison among studies should therefore be made cautiously.

## 4. Common Methods for Assessing Locomotor Condition in Broilers

Assessment of the broiler locomotor system includes both simple functional methods and advanced imaging, biomechanical, and automated monitoring techniques. Assessment of locomotor function, particularly gait and walking ability, is also an important component of broiler welfare assessment because impaired locomotion is directly associated with pain, restricted access to resources, and reduced ability to perform normal behavior [51]. No single method, however, provides a complete assessment of leg health. Observational methods effectively reflect a bird’s functional capacity but may fail to detect subclinical lesions, whereas imaging and laboratory diagnostics identify pathology more accurately but are more difficult to apply at flock level. Consequently, combining functional, structural, and technological indicators is becoming increasingly important [51]. To facilitate direct comparison of their practical applicability, the main characteristics, strengths, and limitations of commonly used assessment methods are summarized in Table 2.

### 4.1. Gait Scoring

Gait scoring remains one of the most commonly used field methods for assessing locomotor function in broilers. The most widely used six-point scale, known as the Bristol gait score, ranges from 0, representing normal gait, to 5, representing complete inability to walk [7,52,53]. Birds are usually observed while moving over a short, level surface, with assessment of symmetry, stability, and ability to take successive steps. Scores of ≥3 indicate marked mobility impairment and a significant welfare concern [54].

The major advantages of this method are its simplicity and direct applicability on the farm. Gait score, however, primarily assesses the functional outcome of pathology rather than its cause. Early bone or cartilage lesions may remain undetected until they affect locomotion. In addition, results depend on observer experience, the method used to stimulate the bird to move, flooring type, age, and body weight [6]. Gait scoring should therefore be regarded as a useful screening indicator rather than a stand-alone diagnostic method.

### 4.2. Standing Ability

Assessment of standing ability is a simple functional test that determines whether a broiler can independently assume and maintain a standing posture and appropriately bear weight on both limbs. Depending on the scoring system used, assessment may include unilateral or bilateral abnormalities, degree of balance impairment, and complete inability to maintain a standing posture [55].

The advantage of this method is the direct assessment of the bird’s capacity to support its own body weight, which is particularly relevant in heavy broilers during the final phase of rearing. Standing ability may reveal severe functional impairment even when detailed gait assessment is difficult to perform. At the same time, the method characterizes the consequence rather than the etiology of the disorder. A bird may have difficulty standing because of bone deformity, joint lesions, pain, muscle weakness, or infectious disease. The result should therefore be interpreted together with gait score, clinical examination, or structural diagnostic assessment [52]. Its greatest value lies in rapid identification of birds whose functionality has already been substantially compromised.

### 4.3. Latency-to-Lie Test

The latency-to-lie test measures how long a broiler remains standing under standardized conditions, most commonly after placement in shallow water. The test is based on the bird’s natural reluctance to sit in water. Birds with good leg function remain standing longer, whereas a shorter latency to lie may indicate pain, fatigue, or reduced ability to support body weight [52].

An advantage of the test is that it provides a quantitative parameter that may be more objective than conventional ordinal gait scoring. However, latency to lie is not a direct test of skeletal disease. Results may be influenced by bird temperament, body weight, water temperature, handling stress, and previous activity. The method also requires standardized conditions, which limits its use in routine assessment of large flocks. Its greatest value may therefore lie in experimental and validation studies, particularly as a complementary method to gait scoring. In the future, it may form part of more comprehensive welfare-assessment systems, although further validation under commercial conditions is required.

### 4.4. Radiographic Examination (X-Ray)

Radiography enables assessment of skeletal structures that are not visible during clinical examination and can reveal deformities, impaired mineralization, and changes within growth plates. It is particularly useful in the diagnosis of tibial dyschondroplasia and assessment of bone geometry, including the tibial plateau angle [23]. It may therefore detect pathology before overt lameness develops.

The major advantage of radiography is the ability to combine clinical assessment with visualization of structural lesions, thereby increasing diagnostic accuracy. However, its use for routine monitoring of large flocks is limited. It requires specialized equipment, appropriate positioning or restraint of the bird, and interpretation by an experienced diagnostician. The cost and time required mean that radiography is used primarily in research, diagnosis of individual cases, or assessment of representative samples. Nevertheless, it remains an important reference method for verifying whether observed gait abnormalities correspond to changes in bone structure.

### 4.5. Gross Post-Mortem Examination

Post-mortem examination enables direct assessment of bones, joints, cartilage, tendon sheaths, and soft tissues and therefore remains one of the most important methods for confirming locomotor lesions. It can reveal, among other abnormalities, thickening of the growth plate in tibial dyschondroplasia, joint effusion, necrosis, inflammatory lesions, and damage to periarticular structures [23,53]. Samples may subsequently be used for histopathological, microbiological, or biomechanical analyses.

The major advantage of this method is that it enables direct linkage of functional impairment with a specific anatomical pathology. At the same time, it is an endpoint method: it does not allow longitudinal monitoring of the same individual and requires euthanasia or the use of post-mortem material. It is therefore usually applied to a representative subset of birds [45]. In welfare research, combining post-mortem findings with earlier gait scores is particularly valuable because it helps determine how accurately observed locomotion reflects the true severity of pathological lesions.

### 4.6. Biomarkers

Biomarkers may enable detection of metabolic, inflammatory, or bone-remodeling changes before visible locomotor signs appear. Measurement of parameters in blood, tissues, or other biological materials may potentially monitor processes related to bone formation and resorption, inflammation, or cartilage damage [23]. Representative examples include markers of bone formation and mineral metabolism, such as alkaline phosphatase, osteocalcin, calcium, phosphorus, and vitamin D metabolites; markers of bone resorption, including tartrate-resistant acid phosphatase; inflammatory indicators such as interleukin-6, tumor necrosis factor-α, and C-reactive protein; and markers associated with cartilage matrix turnover or damage, including collagen-derived degradation products. Metabolomic and microbiome-based approaches may additionally identify novel candidate biomarkers associated with skeletal disorders such as tibial dyschondroplasia [56].

Biomarkers are therefore particularly valuable as tools for the early detection of subclinical changes that are not identified by conventional gait scoring. Their use may also enable objective assessment of disease dynamics and the effectiveness of nutritional or environmental interventions [56,57]. However, most biomarkers lack specificity for individual locomotor disorders and may also be influenced by age, growth rate, nutrition, systemic inflammation, stress, or other diseases. In addition, sample collection and laboratory analyses increase costs and limit routine flock-level application. Biomarkers should therefore currently be regarded primarily as complementary tools rather than alternatives to functional, imaging, or pathological assessment.

### 4.7. Infrared Thermography (IRT)

Infrared thermography is a non-contact method for recording body-surface temperature and may identify local thermal changes associated with altered blood flow, inflammation, or tissue damage. In broilers, it can be applied to the feet, joints, and other limb regions without restraining or euthanizing the bird [23]. One of its best-described applications is the assessment of footpad dermatitis, in which lesion development may be associated with altered surface temperature [3]. However, footpad dermatitis should be considered a cutaneous condition that can secondarily affect locomotion through pain and reduced willingness to move, rather than a direct indicator of skeletal or joint pathology.

For locomotor disorders specifically, the potential value of infrared thermography lies in identifying localized thermal asymmetries or inflammatory changes in joints and limb regions that may warrant further clinical or imaging examination. However, such thermal abnormalities are non-specific and cannot distinguish reliably among skeletal, articular, soft-tissue, or infectious causes of locomotor impairment. Results are also influenced by ambient temperature, humidity, camera distance, feather cover, and bird activity. Therefore, thermography should be regarded primarily as a screening tool rather than a stand-alone diagnostic method for locomotor disease.

### 4.8. Force Plates

Force plates enable objective measurement of ground reaction forces generated by the limbs during standing or walking. These measurements allow assessment of the magnitude and distribution of limb loading and may reveal asymmetrical weight bearing even when impairment is not yet clearly visible during observation. A painful, weakened, or deformed limb is typically loaded less, with part of the body weight shifted to the contralateral side [52]. Unlike gait scoring, this method provides quantitative biomechanical data and reduces the influence of subjective observer assessment. It can therefore reveal subtle changes in gait pattern and serve as a valuable tool for validating other methods. Its limitation is the need to guide the bird across an appropriately prepared measurement platform and obtain correct limb contact with the sensing surface. Bird behavior, walking speed, and foot placement may substantially influence the results. Consequently, force plates remain primarily a research tool, although they may play an important role in the development of objective algorithms for automated gait assessment.

### 4.9. Camera-Based and Sensor-Based Monitoring

Camera-based and sensor-based systems are increasingly investigated for automated assessment of broiler locomotion. In camera-based approaches, computer-vision and image-analysis algorithms are used to extract locomotor features from images or video recordings, including bird position, movement speed, stride characteristics, lying time, movement symmetry, and lateral body sway. Pose-estimation models may additionally track anatomical landmarks such as the head, hock joints, and feet to characterize gait patterns [52]. Sensor-based approaches, in contrast, rely on non-imaging devices, such as wearable or environmental sensors, to quantify activity, location, movement dynamics, or spatial behavior.

The principal advantage of these technologies is their potential for continuous, non-invasive monitoring of large groups of birds without repeated manual handling. However, their current practical applicability remains constrained by several important limitations. Individual identification is difficult in large commercial flocks, particularly when birds are visually similar and frequently occlude one another at high stocking densities. Camera performance may also be affected by lighting conditions, litter characteristics, bird density, camera position, and housing-system design. Consequently, algorithms developed under controlled or farm-specific conditions may not perform equally well across farms, camera configurations, genotypes, bird ages, housing systems, or environmental conditions. External validation under diverse commercial conditions is therefore essential before such systems can be considered suitable for routine locomotor assessment.

A further challenge is the biological interpretation of reduced activity. Decreased movement may indicate locomotor pain or lameness, but it may also result from resting behavior, heat stress, lighting schedules, stocking density, social interactions, or other health problems. Automated systems therefore cannot assume that reduced activity is equivalent to impaired locomotor function. Validation against expert gait scoring, clinical examination, and, where possible, structural or pathological findings remains essential. Sensitivity and specificity should also be reported before automated classifications are considered suitable for routine welfare surveillance.

At present, some technologies are useful for research, continuous activity monitoring, and development of early-warning indicators, whereas fully validated systems for autonomous diagnosis of locomotor disorders under diverse commercial conditions remain limited. The most promising approach is therefore likely to combine camera-based or sensor-based data with established functional and clinical measures rather than to replace conventional assessment entirely.

## 5. Conclusions

Locomotor health in modern broilers should be considered the outcome of interacting genetic, developmental, nutritional, biomechanical, environmental, and infectious factors rather than the consequence of a single production trait. Although considerable progress has been made in breeding, nutrition, housing, and locomotor assessment, important uncertainties remain regarding the relative contribution and interaction of these factors under current commercial conditions.

Future research should therefore focus on several specific priorities. First, longitudinal studies are needed to determine the temporal relationships among rapid growth, body conformation, reduced activity, pain, and the development of structural locomotor lesions, thereby distinguishing causes from consequences more clearly. Second, contemporary commercial studies should quantify the production consequences of locomotor impairment using standardized measures of growth, feed intake, feed efficiency, mortality, culling, carcass condemnation, and processing losses. Third, genetic studies should further evaluate whether continued improvement in growth and breast-muscle yield can be combined with skeletal robustness and functional mobility without unfavorable correlated responses.

Further work is also required to define nutritional strategies according to genotype, growth trajectory, and nutrient bioavailability rather than relying on generalized nutrient recommendations. Similarly, environmental and enrichment interventions should be evaluated across different stocking densities, genotypes, and commercial housing systems to determine which strategies produce consistent improvements in locomotor function.

Finally, automated locomotor-monitoring technologies require external validation under commercial conditions. Future studies should establish their sensitivity and specificity relative to expert gait scoring, clinical examination, and pathological findings, and determine whether automated systems can reliably distinguish lameness-related inactivity from normal resting behavior or activity changes caused by environmental factors.

From a practical welfare perspective, current EFSA recommendations indicate that stocking density should not exceed 11 kg/m^2^ when the objective is to support normal behavior, locomotor ability, and health [51]. However, stocking density should not be considered independently of other environmental conditions. An activity-supporting environment should include dry and friable litter, appropriate light–dark cycles, adequate thermal conditions and ventilation, easy access to feed and water, and environmental complexity that encourages voluntary movement, such as elevated platforms or other functional enrichment.

Progress in these areas will be essential for developing production systems in which locomotor welfare and production efficiency are evaluated together rather than as competing outcomes. Future studies should also evaluate multimodal systems integrating camera- and sensor-based data with gait scores, clinical indicators, and structural findings, while developing robust individual-level tracking and prospectively validating early-warning models under commercial farm conditions.

## Figures and Tables

**Table 1:** Comparative characteristics of the major locomotor disorders in broiler chickens.

Disorder	Main Origin/ Pathogenesis	Typical Age/Stage	Main Anatomical Site	Clinical/Subclinical Presentation	Welfare Impact	Main Diagnostic Approach
Valgus–varus deformity (VVD)	Developmental/biomechanical	Growing broilers; may become more evident as body mass increases	Distal tibiotarsus; may also involve the tarsometatarsus	Abnormal limb alignment (valgus or varus), altered gait; severity increases with biomechanical loading	Moderate to severe, depending on deformity and resulting mobility impairment	Clinical examination, gait assessment, post-mortem examination
Tibial dyschondroplasia (TD)	Developmental/metabolic; impaired chondrocyte maturation, vascularization and mineralization	Rapid growth phase	Growth plate of the proximal tibiotarsus	May remain subclinical; severe cases show unstable gait, reluctance to move and use of wings for support	Moderate to severe	Radiography, post-mortem examination, histopathology
Infectious arthritis and tenosynovitis	Infectious/inflammatory	Variable; acute, chronic or subclinical forms may occur	Hock region, joints, digital flexor and gastrocnemius tendon sheaths	Pain, swelling, restricted joint mobility and lameness; subclinical forms may occur	High, primarily due to pain and progressive restriction of mobility	Clinical examination, microbiological testing, post-mortem examination
Bacterial chondronecrosis with osteomyelitis (BCO)	Infectious + biomechanical; bacterial colonization of mechanically damaged osteochondral tissue	Usually becomes clinically important during rapid later growth	Proximal femur and tibia, cartilage, growth plates and bone tissue	Lameness, reluctance to move, use of wings for support; advanced cases may lose the ability to walk	High	Post-mortem examination, bacteriology, imaging where appropriate
Osteochondrosis	Developmental/vascular/biomechanical; disturbance of endochondral ossification	Young, rapidly growing birds	Growth cartilage; cervical/thoracic vertebrae and femoral epiphysis may be affected	May initially be subclinical; shortened stride, reluctance to walk, increased sitting and pain may develop	Moderate, potentially high in clinically affected birds	Post-mortem examination, histopathology, imaging
Rickets	Metabolic/nutritional; impaired mineralization associated mainly with Ca, P and vitamin D3 imbalance or availability	Young, growing birds	Generalized developing skeleton and growth plates	Poor mineralization, soft bones, deformities and fractures; subclinical forms may occur	Moderate to severe	Clinical examination, post-mortem bone assessment, dietary/mineral evaluation, histology where required
Fractures	Traumatic/biomechanical/secondary to underlying skeletal disease	Variable; risk may increase with greater body mass and underlying pathology	Primarily long bones; site depends on trauma or predisposing lesion	Acute pain, impaired weight bearing and reduced mobility	Severe	Clinical examination, radiography, post-mortem examination

**Table 2:** Comparative characteristics of methods used to assess locomotor condition in broiler chickens.

Method	Objectivity	Invasiveness	Relative Cost	Labor Requirement	Flock-Level Suitability	Detection of Subclinical Disease	Major Limitations
Gait scoring	Moderate	Non-invasive	Low	Moderate	Moderate	Low	Observer-dependent; limited sensitivity for early or subclinical lesions; scoring systems may differ among studies
Latency-to-lie test	Moderate	Minimally invasive	Low	Moderate	Low–moderate	Low–moderate	Time-consuming; influenced by motivation, fatigue, age, and test conditions
Standing ability	Moderate	Non-invasive	Low	Low–moderate	Moderate	Low	Limited specificity; influenced by body weight, motivation, and general weakness
Radiography	High	Minimally invasive	High	High	Low	High	Requires specialized equipment and bird handling; limited feasibility for large-scale flock screening
Post-mortem examination	High	Terminal/invasive	Moderate	High	Low	High	Cannot be used for longitudinal monitoring; only provides information after death or culling
Biomarkers	High	Minimally invasive to invasive	Moderate–high	Moderate	Low	Potentially high	Limited specificity of many markers; lack of fully validated locomotor-specific biomarkers and reference ranges
Infrared thermography	Moderate–high	Non-invasive	Moderate–high	Low–moderate	Moderate–high	Potentially moderate	Affected by ambient temperature, humidity, feather coverage, distance, and image-acquisition conditions
Force plates	High	Non-invasive	High	High	Low	Moderate–high	Specialized equipment; controlled measurement conditions; limited throughput and field scalability
Camera/sensor systems	High	Non-invasive	High initial cost	Low after implementation	High	Potentially high	Requires validation; affected by occlusion, individual identification, algorithm performance, and system standardization

## Data Availability

No new data were created or analyzed in this study. Data sharing is not applicable to this article.
